# Using Single Cell Transcriptomics to Elucidate the Myeloid Compartment in Pancreatic Cancer

**DOI:** 10.3389/fonc.2022.881871

**Published:** 2022-05-19

**Authors:** Padma Kadiyala, Ahmed M. Elhossiny, Eileen S. Carpenter

**Affiliations:** ^1^ Department of Immunology, University of Michigan, Ann Arbor, MI, United States; ^2^ Department of Computational Medicine and Bioinformatics, University of Michigan, Ann Arbor, MI, United States; ^3^ Department of Intenal Medicine, Division of Gastroenterology, Michigan Medicine, University of Michigan, Ann Arbor, MI, United States

**Keywords:** PDAC, single cell, tumor microenvironment, MDSC, myeloid, TAM

## Abstract

Pancreatic ductal adenocarcinoma (PDAC) is a dismal disease with a 5-year survival rate of 10%. A hallmark feature of this disease is its abundant microenvironment which creates a highly immunosuppressive milieu. This is, in large part, mediated by an abundant infiltration of myeloid cells in the PDAC tumor microenvironment. Consequently, therapies that modulate myeloid function may augment the efficacy of standard of care for PDAC. Unfortunately, there is limited understanding about the various subsets of myeloid cells in PDAC, particularly in human studies. This review highlights the application of single-cell RNA sequencing to define the myeloid compartment in human PDAC and elucidate the crosstalk between myeloid cells and the other components of the tumor immune microenvironment.

## Introduction

Pancreatic cancer (PDAC) remains a deadly disease and is notoriously challenging to treat with a 5-year survival rate of 10% (1). While the only curative treatment is complete surgical resection, fewer than 20% of patients are eligible due to advanced stages of disease, making medical therapies the mainstay for most PDAC patients. Unfortunately, while PDAC initially responds well to the standard combination therapies of FOLFIRINOX or gemcitabine/nab-paclitaxel, most patients progress due to chemoresistance, leading to poor outcomes (2). As the mechanisms of tumor progression and chemoresistance are multifactorial and poorly understood, there is an unmet need for the development of better treatment strategies. Growing evidence demonstrates that the tumor microenvironment (TME) is a vital component in the pathogenesis of PDAC and plays an essential role in tumor progression, invasion, and therapeutic resistance (3). Desmoplastic stroma comprises up to 80% of total tumor volume and largely consists of immune cells, fibroblasts, and acellular collagens (4). In particular, the accumulation of myeloid cells in the TME drives immune suppression (5–7). Previous studies have shown that targeting the myeloid compartment within PDAC tumors in murine models led to increased cytotoxic T cell activity, decreased regulatory T cell activity, shrinkage of tumors, and improved survival (5–7); however, clinical trials targeting myeloid cells have failed or only partially recapitulate results from preclinical models in a subset of patients (8, 9). This highlights the lack of fidelity in using preclinical murine models for human PDAC.

**Figure 1:**
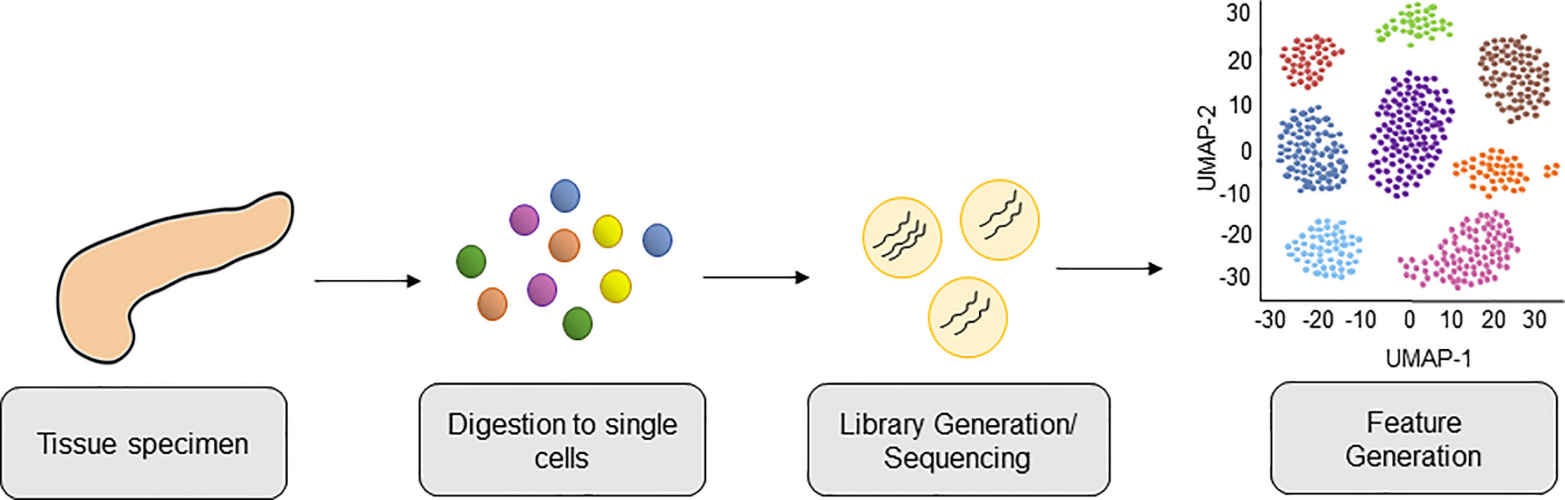
Workflow of Single-Cell RNA-sequencing PDAC tissue from patients. PDAC tissue is collected from patient donors and digested into a single cell suspension of live cells. Cells are lysed, cellular mRNA captured, and cDNA libraries are generated and subjected to high-throughput sequencing. This is followed by bioinformatics analysis, including downstream feature generation and visualization of cells clusters by Uniform Manifold Approximation and Projection (UMAP).

**Figure 2:**
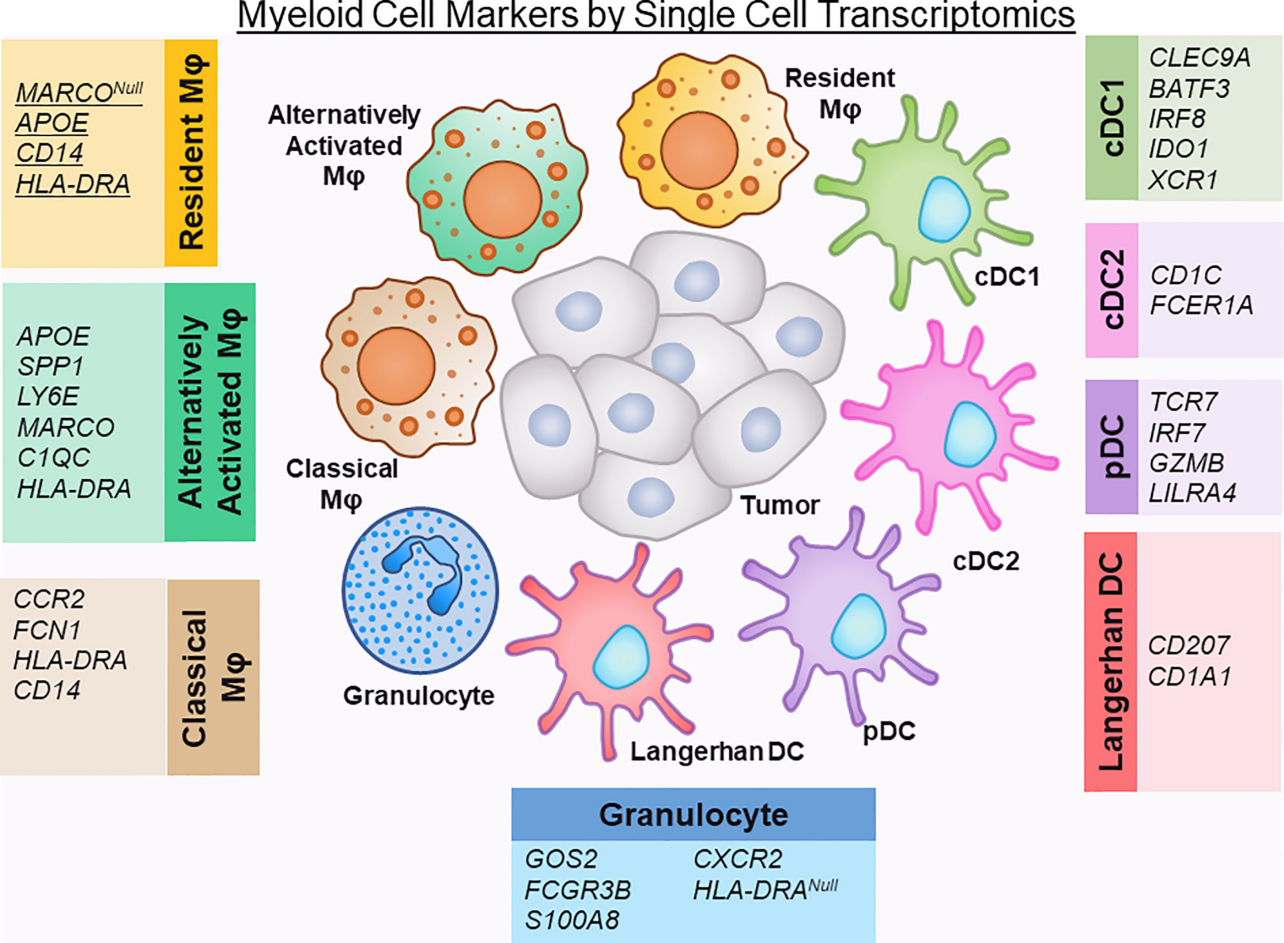
Defining myeloid cell markers by single cell transcriptomics.

Strategies to molecularly profile human pancreatic tumors are thus crucial to help unravel the complexities of human disease. Recently, single-cell RNA sequencing (scRNA-seq) has been shown to provide the analytical power to define cell-specific molecular signatures and map out the interactions of cell types within the TME (10–15) (**Figure 1**). This analytical strategy is capable of characterizing cell types and states in an unbiased manner, and is key to elucidating the behavior of myeloid cells within the PDAC TME. Ultimately, harnessing the power of scRNA-seq technology can help unravel the intricacies of these cell-cell interactions in human PDAC and lead to the development of novel therapies targeting the microenvironment to improve outcomes for this dismal disease. In this review we highlight the latest progress made in classifying myeloid cell compartment in PDAC by unbiased single-cell analysis (**Figure 2**).

## The Role of Myeloid Cells in PDAC

Within the immune compartment of the PDAC microenvironment, myeloid cells have been shown to be key regulators of immunosuppression and strong correlators to poor clinical outcomes (16, 17).They are abundant in the TME by way of myeloid-promoting cytokines such as CSF-1 and CCL-2 (18, 19). Perhaps their most well-known role in PDAC is their ability to mitigate anti-tumor effector T cell function through the release of cytokines that are immunosuppressive and in turn recruit other cells known to further dampen cytotoxic immune responses, such as T regulatory cells (20). Myeloid cells also mediate the expression of immune checkpoint ligands on tumor cells as another mechanism of immune evasion (21).

Myeloid cells are also known to play other roles that are independent of T cell responses. Tumor-infiltration of these cells is critical for PDAC initiation, as they directly promote the formation and maintenance of preneoplastic lesions through factors including EGF ligand and PDGF in murine models of PDAC (22, 23).

Interestingly, myeloid cells have been shown to directly enhance chemoresistance in PDAC tumor cells *in vitro* using indirect co-culture assays, implicating soluble factors as mediators (24, 25). It has been previously shown that conditioned media from tumor-educated bone marrow-derived macrophages confers chemoresistance to gemcitabine *in vitro*, specifically through pyrimidine release in myeloid cells (25).

Studies have also suggested that myeloid cells play a vital role in the pre-metastatic niche as a precursor colonizer to metastatic sites that allow for a favorable environment for tumor cell seeding and growth (26, 27). Recently, a new role of myeloid cells was uncovered in mouse models linking myeloid cell invasion into the central nervous system leading to cachexia symptoms in PDAC (28).

While these studies provide information about the behavior of myeloid cells and their response to numerous environmental stimuli to promote PDAC pathogenesis, the bulk of these studies were performed in preclinical murine models, with limited correlation in human studies. Recent single cell studies on human tumor tissue have allowed for a better understanding of the transcriptional diversity and putative function of myeloid cells in human disease.

## PDAC Myeloid Cell Subtypes in Single Cell Transcriptomics

Previously, characterization of the myeloid compartment within TME in human studies was limited to immunostaining and flow cytometric techniques, while more in-depth study of transcriptional networks involved in the myeloid compartment of PDAC tumors could only be determined through deconvolution methods (29) (See **Table 1**). However, with the advent of multidimensional single-cell and spatial techniques, we now know that the myeloid compartment in PDAC has a complex heterogeneity. It is important to note that there is a diverse array of different transcriptomic patterns of myeloid cells across different cancer types, and care should be taken before generalizations are made regarding the myeloid transcriptome of PDAC, which varies from other solid tumor types (30). **Table 2** summarizes significant contribution to human single cell sequencing on PDAC to date.

**Table 1:** Advantages and disadvantages of technologies used for classifying myeloid compartments in pancreatic cancer.

Technique	Advantages	Disadvantages
Single Cell RNA Sequencing	Distinguish cell types at high-resolution in an unbiased manner Identify states of cells in different development, differentiation, and cell cycle states in tissues Gene expression profiles could be used to computationally map the cell trajectory	Requires processing of fresh tissue Determining spatial distribution of the cell type is not possible. Read dropout and false discoveries
Flow Cytometry	Identity frequency and activate state of the cells Characterize heterogenous cell populations. Cell populations can be sorted Results can be obtained in a short time.	Cannot classify new cell types and their states in an unbiased manner Cell morphology cannot be visualized Cell populations with similar marker expression cannot be differentiated Fluorophore signal spillover
Immunohistochemistry	Identify localization of the protein in tissue Acquire information about tissue architecture, size, and shape of the cells Results can be obtained in days	Restricted to limited number of markers Immunolabelling depends on the specificity of primary antibodies Semi-quantitative approach

**Table 2:** All significant published studies that have provided new single cell RNA sequencing datasets in pancreatic cancer.

Year	Study	Reference
2019	Elyada, E. et al.-Single cell sequencing of 6 treatment-naive PDAC tumors and 2 adjacent normal pancreas tissue.	(10)
2019	Peng, J. et al.-Single cell sequencing of 24 treatment-naive PDAC tumors and 11 normal pancreas tissue.	(11)
2019	Bernard, V. et al.-Single cell sequencing of 2 PDAC and 4 IPMN specimens.	(14)
2020	Steele, N.G. et al.-Single cell sequencing of 16 treatment-naïve PDAC tumors from surgical resections and fine needle biopsies as well as 3 adjacent normal pancreas tissue.	(13)
2020	Hwang, W.L. et al.-Single nucleus sequencing of frozen archival surgically resected tumors from 26 patients, 11 treated and 15 treatment naïve	(15)
2021	Raghavan, S. et al.-Single cell sequencing of core needle biopsies from 17 untreated and 6 treated liver metastasis	(12)
2021	Kemp, S.B. et al.- Single cell sequencing of 2 treated and 3 treatment-naive liver metastasis	(26)
2021	Cheng, S. et al.-Single cell sequencing of 6 treatment-naïve PDAC tumors and 3 adjacent normal pancreas tissue	(30)
2021	Zhou, D.C. et al.-Single cell sequencing of 7 treatment-naïve, 14 treated PDAC tumors and 4 adjacent normal pancreas tissue.	(31)

### Myeloid-Derived Suppressor Cells (MDSCs)

MDSCs are a heterogeneous population of immature myeloid cells that have the ability to suppress adaptive T-cell immunity, resulting in mitigation of cytotoxic anti-tumor activity (32). In PDAC patients, levels of MDSCs in the peripheral blood correlate with stage of disease progression (33, 34). Their primary role in inhibiting anti-tumor immunity of effector T Cells is accomplished *via* direct and indirect mechanisms, including crosstalk with other immunosuppressive cell types. MDSCs have been shown to influence regulatory T cells, dendritic cells, and TAMs (tumor associated macrophages), thereby promoting tumor immunotolerance (20, 33, 35, 36). In a subset of cancers, temporal decline in MDSC levels with treatment has correlated with better survival (37–40). Further studies are needed to determine whether changes in MDSC levels over time bear clinical relevance in PDAC.

It is important to note that because there is no consensus set of protein markers for MDSCs, and an even more poorly-defined transcriptomic signature, MDSCs have yet to be identified within PDAC single cell datasets. MDSCs are comprised largely of monocytic MDSCs and granulocytic MDSCs in a nomenclature to mimic their normal counterparts. In murine model of breast cancer, one group arrived at a transcriptomic signature for monocyte MDSCs and granulocytic MDSCs; the signature for monocytic MDSCs did not translate to any population in a human dataset for breast cancer, but the granulocytic signature was enriched in breast cancer-associated neutrophils (41). More studies need to be done in PDAC to identify whether the MDSCs transcriptionally represent a subpopulation unique from their normal myeloid counterparts.

### Tumor Associated Macrophages (TAMs)

Traditionally, the macrophages in PDAC have been oversimplified into a proinflammatory/antitumorigenic phenotype (M1) and an anti-inflammatory/protumorigenic phenotype (M2) (42), which does not accurately reflect the *in vivo* heterogeneity seen in human tumors. Indeed, traditional M1 and M2 markers do not dichotomize macrophage populations within single cell datasets and often canonical markers for both are found within the same cell (43).

Recent single cells studies on human PDAC tumor tissue have reclassified these macrophages into subtypes that more accurately represent their *in vivo* state, namely resident, classical, alternatively-activated TAMs (10, 13). Alternatively activated macrophages express *APOE*, *SPP1, LY6E*, and the macrophage scavenger receptor *MARCO*, while resident TAMs lack *MARCO* expression. Of note, in other solid tumor cancers, *MARCO* expression has been associated with a pro-tumor, immunosuppressive phenotype of macrophage activation (44, 45). *APOE* has recently been found in mouse models to promote an immunosuppressive microenvironment in PDAC through NF-kB signaling (46). Classical TAMs express less of a committed macrophage transcriptomic phenotype (lower expression of CD68 and HLA-DR) and suggest an intermediary state of monocytes migrating from blood to tissue and maturing into macrophages (30, 43).

Another classification system has also emerged whereby TAMs are subdivided into FCN1+ TAMs (monocyte-like, and akin to classical TAMs), SPP1+ TAMs, or C1QC+ TAMs (12, 30). Together, SPP1+ and C1QC+ TAMs overlap with resident and alternatively-activated TAMs in the previous classification system. Of note, complement-high macrophages (C1QA, C1QB, and TREM2) may play an important role is establishing the premetastatic niche, as these particular macrophages have been found to be further enriched in liver metastatic lesions compared to primary tumors in human PDAC (26). C1QC+ TAMs have been found to be associated with basal-like tumors where T cells are notably sparse (12).

### Neutrophils

Neutrophils are abundant in the TME of PDAC, and have been shown to have dual tumor-promoting and anti-tumorigenic functions (47, 48). Despite this, most single cell transcriptomic studies do not identify a neutrophil population, with possible causes for this underrepresentation including the techniques used to process and purify cells and the difficulty in capturing adequate RNA reads for this particular cell type (11, 12, 14, 30). Of note, in the dataset by Elyada, et al., it was noted that neutrophil markers were present within the myeloid dataset, but these particular genes were intermixed within the monocyte/macrophage populations (10). Steele and colleagues were able to identify a separate granulocytic population in their dataset that was defined by expression of *FCGR3B (CD16), S100A8, CXCR2*, and absence of *HLA-DRA* (13). Further studies need to be performed to dissect whether heterogeneity in the neutrophil population can be captured with single-cell transcriptomics.

### Dendritic Cells

Dendritic Cells are a specialized group of antigen-presenting cells that play a key role in initiating both innate and adaptive immune responses (49). The relative absence of dendritic cells in the PDAC TME has been linked to dysfunctional immune surveillance in PDAC, with poor T cell responses to tumor neoantigens (50). Single cell transcriptomic studies have identified several subsets of dendritic cells: conventional (cDC), plasmacytoid (pDC), and Langerhans-like. The cDCs can be further subdivided into cDC1 (Type 1), which cross‐present antigens *via* MHC class I to activate CD8^+^ T cells, and cDC2 (type 2), which produce high levels of IL‐12 and are potent activators of CD4 T helper responses (51). By single cell sequencing, dendritic cells have been named using different defining markers, likely due to technical differences in specimen processing and read depth. cDC1s have been identified previously by expression of *CLEC9A, BATF3, IRF8, IDO1* (10). cDC2 have been characterized by expression of CD1C, FCER1A (14). Additionally, XCR1, a chemokine receptor, is selectively expressed on cDC1s and also has been used to subset cDC1 cells (12). Plasmacytoid dendritic cells (pDCs), in comparison to cDCs have poor antigen-presenting function, but are potent producers of type 1 interferons (51). They have been defined in single cell transcriptomics by *TCR7, IRF7* and *GZMB* positivity, as well as *LILRA4* positivity (10, 12, 14). Langerhans-like DC, which are immature dendritic cells that mediate immune tolerance, are defined by *CD207* and *CD1A* expression (10).

## Leveraging Single Cell Studies for the Myeloid Compartment in PDAC

### Myeloid Expression of Checkpoints

Immunotherapy has notoriously been unsuccessful in improving outcomes in PDAC (52, 53). The reason for this has, in part, been elucidated through single cell studies showing abundant and varied expression of immune checkpoints across the myeloid compartments. For example, TAMs have upregulated *LGALS* (ligand for TIM3) as well as its binding counterpart *TIM3*, *PVR* (ligand for *TIGIT*), *and HLA-DRA* (13, 31). Certain subsets of dendritic cells also had elevated expression of immune checkpoint ligands, suggesting a potential immunosuppressive role (13). Of note, wide heterogeneity of immune checkpoint expression within the myeloid compartment was observed between patients, suggesting the need for a precision pipeline in identifying appropriate immunotherapeutic regimens for each patient (13).

### Myeloid Crosstalk Within the Tumor Microenvironment

Prior to single cell studies, several mediators of crosstalk involving the myeloid compartment of the TME have been identified, including the CSF1/CSF1R axis, the CCL2/CCR2 axis, and the ELR+ chemokine/CXCR2 axis (7, 18, 19). With the advent of singe cell signaling, one useful tool to identify putative cross-talk interactions in single cell datasets is the use of mapping algorithms of known ligand-receptor interactions across different cell types (54). Mapping these interactions are a boon in the study of the TME, which relies on the complex interplay between tumor and non-tumor cells. Using this technique, new putative ligand-receptor interactions between myeloid/epithelial cells and myeloid/lymphocytes have been identified. In the dataset published by Lee et al., myeloid populations were the most well-connected to epithelial cells, with notable interactions including MIF/CD74 (HLA-DR allele), and APP/CD74 (14). Steele and colleagues also reported multiple interactions between the myeloid and T cell compartment, including ICOS/ICOSLG, SIRPA/CD47, and TIGIT/PVR (13).

These data are in concordance with previous studies showing that myeloid cells are major drivers of the immunosuppressive TME, and provide insights for potential new combination immunotherapy trials in PDAC.

Another recent area of interest in TME crosstalk is the myeloid/fibroblast axis. Using a combination of functional studies and single cell analysis, a recent murine PDAC study demonstrated that hypoxia inducible factor signaling in cancer-associated fibroblasts drives CD86 and PDL1 expression on tumor associated macrophages (55) to dampen anti-tumor immune responses. Similarly, while the TGFβ signaling axis has also been implicated as a key modulator of regulatory T cells and fibroblast crosstalk in the microenvironment (56–58), its axis has also been shown recently to influence myeloid cell activity in PDAC. Both functional studies with patient-derived organoids and human single cells studies confirm that TGFβ ligand is produced by tumor epithelial cells and is associated with the more aggressive basal subtype of PDAC (10, 12, 57). In murine studies, TGFβ was found to decrease the proportion of MDSCs in liver metastasis and increase the expression of PD-L1^High^ TAMs. Additionally, in correlative human bulk tumor sequencing studies TGFβ was found to be associated with an increased TAM signature (59). As TGFβ signaling has gained recent traction in cancer-associated fibroblast polarization (57), further studies are needed to determine if the role of TGFβ in myeloid cells is direct or involves the fibroblast compartment as an intermediary.

## Discussion

Single cell transcriptomic technology has shed much-needed light on the heterogeneity and function of the myeloid compartment in human PDAC. While pre-clinical murine models have dominated the field in the study of the tumor immune microenvironment, results from these studies have led to an oversimplification of the myeloid cell types and have resulted in identifying targets that thus far have had mixed patient outcomes (8, 9, 53). The patient heterogeneity in drug response of these clinical trials is supported by single-cell studies, which highlight the inter-patient heterogeneity of the myeloid compartment. In many of the trials reviewed above, a small subset of patients had some response to the given immunotherapeutic strategy, suggesting that a precision medicine-based platform is needed which can match therapy to each tumor’s microenvironmental characteristics. While there is no such tool in place to tailor these therapies, single-cell transcriptomics bring a promising avenue for both biomarker and therapeutic discovery.

One caveat to note is that the technique of single cell transcriptomics is not without its own flaws, which include variation in tissue acquisition and processing, read “dropout”, and, unfortunately, false discoveries (60, 61). Therefore, validation of gene expression through complementary techniques such as multiplex immunofluorescence or mass cytometry is necessary (**Table 1**). Furthermore, putative interactions and identified signaling networks should be investigated with further functional studies using *in vitro* or *in vivo* systems.

Another limitation to single cell RNA sequencing is that spatial data is not preserved, and validating targets via immunostaining can be laborious. Indeed, work using multiplex immunofluoresence has shown that immune cell localization of myeloid cells within the tumor had important clinical significance in PDAC patients (62). Recent developments in spatial transcriptomics and multiplex staining can add a crucial dimension to identifying cell subtypes in the TME and validating putative crosstalk between cells (16, 63). Alternatively, machine learning has been leveraged with multiplexed immunofluorescence and whole-slide imaging for tissue segmentation and classification (64, 65). Recent bioinformatics pipelines are actively working to integrate these multi-dimensional datasets for a seamless approach and yield new insights on myeloid cells in the TME (66–68).

In conclusion, PDAC remains a deadly disease with an urgent need to find new and better therapies. Targeting the myeloid compartment of the TME is a promising avenue to pursue; although given the complexities of these cells shown by single cell studies, single-agent immunotherapy is likely not sufficient and combinatorial approaches may be required. One exciting avenue to apply single-cell transcriptomics is through the study of tumor tissue longitudinally throughout the course of disease and therapy treatment, as myeloid cells have been shown in preclinical studies to play a major role in the development of chemoresistance (24, 25). Leveraging this technique to comprehensively study the immune microenvironment in the treatment-naïve and post-treatment states may provide new insights to the role of the TME in the development of chemoresistance and ultimately identify new pathways to target in this dismal disease.

## Author Contributions

All authors participated in writing the manuscript, with EC taking the lead in organizing and compiling the final document. PK and EC illustrated **Figures 1**, **2**. All authors contributed to the article and approved the submitted version.

## Funding

This work was supported by the American College of Gastroenterology Career Development Award and the VA BLR & D Career Development Award to EC; T32-AI007413 to PK.

## Conflict of Interest

The authors declare that the research was conducted in the absence of any commercial or financial relationships that could be construed as a potential conflict of interest.

## Publisher’s Note

All claims expressed in this article are solely those of the authors and do not necessarily represent those of their affiliated organizations, or those of the publisher, the editors and the reviewers. Any product that may be evaluated in this article, or claim that may be made by its manufacturer, is not guaranteed or endorsed by the publisher.

## References

[1] Siegel RL Miller KD Jemal A. Cancer Statistics, 2018. CA Cancer J Clin (2018) 68(1):7–30. doi 10.3322/caac.21442 29313949

[2] Zeng S Pottler M Lan B Grutzmann R Pilarsky C Yang H. Chemoresistance in Pancreatic Cancer. Int J Mol Sci (2019) 20(18):4504. doi 10.3390/ijms20184504 PMC6770382 31514451

[3] Turley SJ Cremasco V Astarita JL. Immunological Hallmarks of Stromal Cells in the Tumour Microenvironment. Nat Rev Immunol (2015) 15(11):669–82. doi 10.1038/nri3902 26471778

[4] Erkan M Hausmann S Michalski CW Fingerle AA Dobritz M Kleeff J. The Role of Stroma in Pancreatic Cancer: Diagnostic and Therapeutic Implications. Nat Rev Gastroenterol Hepatol (2012) 9(8):454–67. doi 10.1038/nrgastro.2012.115 22710569

[5] Candido JB Morton JP Bailey P Campbell AD Karim SA Jamieson T. CSF1R(+) Macrophages Sustain Pancreatic Tumor Growth Through T Cell Suppression and Maintenance of Key Gene Programs That Define the Squamous Subtype. Cell Rep (2018) 23(5):1448–60. doi 10.1016/j.celrep.2018.03.131 PMC5946718 29719257

[6] Karakhanova S Link J Heinrich M Shevchenko I Yang Y Hassenpflug M. Characterization of Myeloid Leukocytes and Soluble Mediators in Pancreatic Cancer: Importance of Myeloid-Derived Suppressor Cells. Oncoimmunol (2015) 4(4):e998519. doi 10.1080/2162402X.2014.998519 PMC4485765 26137414

[7] Nywening TM Belt BA Cullinan DR Panni RZ Han BJ Sanford DE. Targeting Both Tumour-Associated CXCR2(+) Neutrophils and CCR2(+) Macrophages Disrupts Myeloid Recruitment and Improves Chemotherapeutic Responses in Pancreatic Ductal Adenocarcinoma. Gut (2018) 67(6):1112–23. doi 10.1136/gutjnl-2017-313738 PMC5969359 29196437

[8] Okusaka T Furuse J. Recent Advances in Chemotherapy for Pancreatic Cancer: Evidence From Japan and Recommendations in Guidelines. J Gastroenterol (2020) 55(4):369–82. doi 10.1007/s00535-020-01666-y PMC7080663 31997007

[9] Nywening TM Wang-Gillam A Sanford DE Belt BA Panni RZ Cusworth BM. Targeting Tumour-Associated Macrophages With CCR2 Inhibition in Combination With FOLFIRINOX in Patients With Borderline Resectable and Locally Advanced Pancreatic Cancer: A Single-Centre, Open-Label, Dose-Finding, Non-Randomised, Phase 1b Trial. Lancet Oncol (2016) 17(5):651–62. doi 10.1016/S1470-2045(16)00078-4 PMC5407285 27055731

[10] Elyada E Bolisetty M Laise P Flynn WF Courtois ET Burkhart RA. Cross-Species Single-Cell Analysis of Pancreatic Ductal Adenocarcinoma Reveals Antigen-Presenting Cancer-Associated Fibroblasts. Cancer Discov (2019) 9(8):1102–23. doi 10.1158/2159-8290.CD-19-0094 PMC6727976 31197017

[11] Peng J Sun BF Chen CY Zhou JY Chen YS Chen H. Single-Cell RNA-Seq Highlights Intra-Tumoral Heterogeneity and Malignant Progression in Pancreatic Ductal Adenocarcinoma. Cell Res (2019) 29(9):725–38. doi 10.1038/s41422-019-0195-y PMC6796938 31273297

[12] Raghavan S Winter PS Navia AW Williams HL DenAdel A Lowder KE. Microenvironment Drives Cell State, Plasticity, and Drug Response in Pancreatic Cancer. Cell (2021) 184(25):6119–37.e26. doi 10.1016/j.cell.2021.11.0177 34890551 PMC8822455

[13] Steele NG Carpenter ES Kemp SB Sirihorachai V The S Delrosario L. Multimodal Mapping of the Tumor and Peripheral Blood Immune Landscape in Human Pancreatic Cancer. Nat Cancer (2020) 1(11):1097–112. doi 10.1038/s43018-020-00121-4 PMC8294470 34296197

[14] Bernard V Semaan A Huang J San Lucas FA Mulu FC Stephens BM. Single-Cell Transcriptomics of Pancreatic Cancer Precursors Demonstrates Epithelial and Microenvironmental Heterogeneity as an Early Event in Neoplastic Progression. Clin Cancer Res (2019) 25(7):2194–205. doi 10.1158/1078-0432.CCR-18-1955 PMC6445737 30385653

[15] Hwang WL Jagadeesh KA Guo JA Hoffman HI Yadollahpour P Mohan. Single-Nucleus and Spatial Transcriptomics of Archival Pancreatic Cancer Reveals Multi-Compartment Reprogramming After Neoadjuvant Treatment. bioRxiv (2020).

[16] Tsujikawa T Kumar S Borkar RN Azimi V Thibault G Chang YH. Quantitative Multiplex Immunohistochemistry Reveals Myeloid-Inflamed Tumor-Immune Complexity Associated With Poor Prognosis. Cell Rep (2017) 19(1):203–17. doi 10.1016/j.celrep.2017.03.037 PMC5564306 28380359

[17] Diaz-Montero CM Salem ML Nishimura MI Garrett-Mayer E Cole DJ Montero AJ. Increased Circulating Myeloid-Derived Suppressor Cells Correlate With Clinical Cancer Stage, Metastatic Tumor Burden, and Doxorubicin-Cyclophosphamide Chemotherapy. Cancer Immunol Immunother (2009) 58(1):49–59. doi 10.1007/s00262-008-0523-4 18446337 PMC3401888

[18] Sanford DE Belt BA Panni RZ Mayer A Deshpande AD Carpenter D. Inflammatory Monocyte Mobilization Decreases Patient Survival in Pancreatic Cancer: A Role for Targeting the CCL2/CCR2 Axis. Clin Cancer Res (2013) 19(13):3404–15. doi 10.1158/1078-0432.CCR-13-0525 PMC3700620 23653148

[19] Zhu Y Knolhoff BL Meyer MA Nywening TM West BL Luo J. CSF1/CSF1R Blockade Reprograms Tumor-Infiltrating Macrophages and Improves Response to T-Cell Checkpoint Immunotherapy in Pancreatic Cancer Models. Cancer Res (2014) 74(18):5057–69. doi 10.1158/0008-5472.CAN-13-3723 PMC4182950 25082815

[20] Siret C Collignon A Silvy F Robert S Cheyrol T Andre P. Deciphering the Crosstalk Between Myeloid-Derived Suppressor Cells and Regulatory T Cells in Pancreatic Ductal Adenocarcinoma. Front Immunol (2019) 10:3070. doi 10.3389/fimmu.2019.03070 32038621 PMC6987391

[21] Zhang Y Velez-Delgado A Mathew E Li D Mendez FM Flannagan K. Myeloid Cells are Required for PD-1/PD-L1 Checkpoint Activation and the Establishment of an Immunosuppressive Environment in Pancreatic Cancer. Gut (2017) 66(1):124–36. doi 10.1136/gutjnl-2016-312078 PMC5256390 27402485

[22] Zhang Y Yan W Mathew E Kane KT Brannon A 3rd Adoumie M. Epithelial-Myeloid Cell Crosstalk Regulates Acinar Cell Plasticity and Pancreatic Remodeling in Mice. Elife (2017) 6:e27388. doi 10.7554/eLife.27388 28980940 PMC5690281

[23] Kaneda MM Cappello P Nguyen AV Ralainirina N Hardamon CR Foubert P. Macrophage PI3Kgamma Drives Pancreatic Ductal Adenocarcinoma Progression. Cancer Discov (2016) 6(8):870–85. doi 10.1158/2159-8290.CD-15-1346 PMC5091937 27179037

[24] Mitchem JB Brennan DJ Knolhoff BL Belt BA Zhu Y Sanford DE. Targeting Tumor-Infiltrating Macrophages Decreases Tumor-Initiating Cells, Relieves Immunosuppression, and Improves Chemotherapeutic Responses. Cancer Res (2013) 73(3):1128–41. doi 10.1158/0008-5472.CAN-12-2731 PMC3563931 23221383

[25] Halbrook CJ Pontious C Kovalenko I Lapienyte L Dreyer S Lee HJ. Macrophage-Released Pyrimidines Inhibit Gemcitabine Therapy in Pancreatic Cancer. Cell Metab (2019) 29(6):1390–9.e6. doi 10.1016/j.cmet.2019.02.001 30827862 PMC6602533

[26] Kemp SB Steele NG Carpenter ES Donahue KL Bushnell GG Morris AH. Pancreatic Cancer Is Marked by Complement-High Blood Monocytes and Tumor-Associated Macrophages. Life Sci Alliance (2021) 4(6):e202000935. doi 10.26508/lsa.202000935 33782087 PMC8091600

[27] Nielsen SR Quaranta V Linford A Emeagi P Rainer C Santos A. Macrophage-Secreted Granulin Supports Pancreatic Cancer Metastasis by Inducing Liver Fibrosis. Nat Cell Biol (2016) 18(5):549–60. doi 10.1038/ncb3340 PMC4894551 27088855

[28] Burfeind KG Zhu X Norgard MA Levasseur PR Huisman C Buenafe. Circulating Myeloid Cells Invade the Central Nervous System to Mediate Cachexia During Pancreatic Cancer. Elife (2020) 9:e54095. doi 10.7554/eLife.54095 32391790 PMC7253193

[29] Moffitt RA Marayati R Flate EL Volmar KE Loeza SG Hoadley KA. Virtual Microdissection Identifies Distinct Tumor- and Stroma-Specific Subtypes of Pancreatic Ductal Adenocarcinoma. Nat Genet (2015) 47(10):1168–78. doi 10.1038/ng.3398 PMC4912058 26343385

[30] Cheng S Li Z Gao R Xing B Gao Y Yang Y. A Pan-Cancer Single-Cell Transcriptional Atlas of Tumor Infiltrating Myeloid Cells. Cell (2021) 184(3):792–809.e23. doi 10.1016/j.cell.2021.01.010 33545035

[31] Zhou DC Jayasinghe RG Herndon JM Storrs E Mo C-K Wu Y. Spatial Drivers and Pre-Cancer Populations Collaborate With the Microenvironment in Untreated and Chemo-Resistant Pancreatic Cancer. BioRxiv (2021) 2021.01.13.426413. doi 10.1101/2021.01.13.426413

[32] Bronte V Brandau S Chen SH Colombo MP Frey AB Greten TF. Recommendations for Myeloid-Derived Suppressor Cell Nomenclature and Characterization Standards. Nat Commun (2016) 7:12150. doi 10.1038/ncomms12150 27381735 PMC4935811

[33] Xu XD Hu J Wang M Peng F Tian R Guo XJ. Circulating Myeloid-Derived Suppressor Cells in Patients With Pancreatic Cancer. Hepatobiliary Pancreat Dis Int (2016) 15(1):99–105. doi 10.1016/s1499-3872(15)60413-1 26818550

[34] Porembka MR Mitchem JB Belt BA Hsieh CS Lee HM Herndon J. Pancreatic Adenocarcinoma Induces Bone Marrow Mobilization of Myeloid-Derived Suppressor Cells Which Promote Primary Tumor Growth. Cancer Immunol Immunother (2012) 61(9):1373–85. doi 10.1007/s00262-011-1178-0 PMC3697836 22215137

[35] Marigo I Dolcetti L Serafini P Zanovello P Bronte V. Tumor-Induced Tolerance and Immune Suppression by Myeloid Derived Suppressor Cells. Immunol Rev (2008) 222:162–79. doi 10.1111/j.1600-065X.2008.00602.x 18364001

[36] Ugolini A Tyurin VA Tyurina YY Tcyganov EN Donthireddy L Kagan VE. Polymorphonuclear Myeloid-Derived Suppressor Cells Limit Antigen Cross-Presentation by Dendritic Cells in Cancer. JCI Insight (2020) 5(15):e138581. doi 10.1172/jci.insight.138581 PMC7455061 32584791

[37] Okla K Czerwonka A Wawruszak A Bobinski M Bilska M Tarkowski. Clinical Relevance and Immunosuppressive Pattern of Circulating and Infiltrating Subsets of Myeloid-Derived Suppressor Cells (MDSCs) in Epithelial Ovarian Cancer. Front Immunol (2019) 10:691. doi 10.3389/fimmu.2019.00691 31001284 PMC6456713

[38] Gonda K Shibata M Ohtake T Matsumoto Y Tachibana K Abe N. Myeloid-Derived Suppressor Cells are Increased and Correlated With Type 2 Immune Responses, Malnutrition, Inflammation, and Poor Prognosis in Patients With Breast Cancer. Oncol Lett (2017) 14(2):1766–74. doi 10.3892/ol.2017.6305 PMC5529875 28789407

[39] Ai L Mu S Wang Y Wang H Cai L Li W. Prognostic Role of Myeloid-Derived Suppressor Cells in Cancers: A Systematic Review and Meta-Analysis. BMC Cancer (2018) 18(1):1220. doi 10.1186/s12885-018-5086-y 30518340 PMC6280417

[40] Ko JS Rayman P Ireland J Swaidani S Li G Bunting KD. Direct and Differential Suppression of Myeloid-Derived Suppressor Cell Subsets by Sunitinib Is Compartmentally Constrained. Cancer Res (2010) 70(9):3526–36. doi 10.1158/0008-5472.CAN-09-3278 PMC3426924 20406969

[41] Alshetaiwi H Pervolarakis N McIntyre LL Ma D Nguyen Q Rath JA. Defining the Emergence of Myeloid-Derived Suppressor Cells in Breast Cancer Using Single-Cell Transcriptomics. Sci Immunol (2020) 5(44):eaay6017. doi 10.1126/sciimmunol.aay6017 32086381 PMC7219211

[42] Murray PJ. Macrophage Polarization. Annu Rev Physiol (2017) 79:541–66. doi 10.1146/annurev-physiol-022516-034339 27813830

[43] Zhang L Li Z Skrzypczynska KM Fang Q Zhang W O'Brien SA. Single-Cell Analyses Inform Mechanisms of Myeloid-Targeted Therapies in Colon Cancer. Cell (2020) 181(2):442–59.e29. doi 10.1016/j.cell.2020.03.048 32302573

[44] La Fleur L Botling J He F Pelicano C Zhou C He C. Targeting MARCO and IL37R on Immunosuppressive Macrophages in Lung Cancer Blocks Regulatory T Cells and Supports Cytotoxic Lymphocyte Function. Cancer Res (2021) 81(4):956–67. doi 10.1158/0008-5472.CAN-20-1885 33293426

[45] Georgoudaki AM Prokopec KE Boura VF Hellqvist E Sohn S Ostling J. Reprogramming Tumor-Associated Macrophages by Antibody Targeting Inhibits Cancer Progression and Metastasis. Cell Rep (2016) 15(9):2000–11. doi 10.1016/j.celrep.2016.04.084 27210762

[46] Kemp SB Carpenter ES Steele NG Donahue KL Nwosu ZC Pacheco A. Apolipoprotein E Promotes Immune Suppression in Pancreatic Cancer Through NF-kappaB-Mediated Production of CXCL1. Cancer Res (2021) 81(16):4305–18. doi 10.1158/0008-5472.CAN-20-3929 PMC8445065 34049975

[47] Nielsen SR Strobech JE Horton ER Jackstadt R Laitala A Bravo MC. Suppression of Tumor-Associated Neutrophils by Lorlatinib Attenuates Pancreatic Cancer Growth and Improves Treatment With Immune Checkpoint Blockade. Nat Commun (2021) 12(1):3414. doi 10.1038/s41467-021-23731-7 34099731 PMC8184753

[48] Deng J Kang Y Cheng Li X Dai B Katz MH. DDR1-Induced Neutrophil Extracellular Traps Drive Pancreatic Cancer Metastasis. JCI Insight (2021) 6(17):e146133. doi 10.1172/jci.insight.146133 PMC8492346 34237033

[49] Marciscano AE Anandasabapathy N. The Role of Dendritic Cells in Cancer and Anti-Tumor Immunity. Semin Immunol (2021) 52:101481. doi 10.1016/j.smim.2021.101481 34023170 PMC8545750

[50] Hegde S Krisnawan VE Herzog BH Zuo C Breden MA Knolhoff BL. Dendritic Cell Paucity Leads to Dysfunctional Immune Surveillance in Pancreatic Cancer. Cancer Cell (2020) 37(3):289–307.e9. doi 10.1016/j.ccell.2020.02.008 32183949 PMC7181337

[51] Eisenbarth SC. Dendritic Cell Subsets in T Cell Programming: Location Dictates Function. Nat Rev Immunol (2019) 19(2):89–103. doi 10.1038/s41577-018-0088-1 30464294 PMC7755085

[52] Royal RE Levy C Turner K Mathur A Hughes M Kammula US. Phase 2 Trial of Single Agent Ipilimumab (Anti-CTLA-4) for Locally Advanced or Metastatic Pancreatic Adenocarcinoma. J Immunother (2010) 33(8):828–33. doi 10.1097/CJI.0b013e3181eec14c PMC7322622 20842054

[53] Topalian SL Hodi FS Brahmer JR Gettinger SN Smith DC McDermott DF. Safety, Activity, and Immune Correlates of Anti-PD-1 Antibody in Cancer. N Engl J Med (2012) 366(26):2443–54. doi 10.1056/NEJMoa1200690 PMC3544539 22658127

[54] Ramilowski JA Goldberg T Harshbarger J Kloppmann E Lizio M Satagopam VP. A Draft Network of Ligand-Receptor-Mediated Multicellular Signalling in Human. Nat Commun (2015) 6:7866.doi 10.1038/ncomms8866 26198319 PMC4525178

[55] Garcia Garcia CJ Huang Y Fuentes NR Turner MC Monberg ME Lin D. Stromal HIF2 Regulates Immune Suppression in the Pancreatic Cancer Microenvironment. Gastroenterol (2022) S0016-5085(22):00154–8. doi 10.1053/j.gastro.2022.02.024 PMC9278556 35216965

[56] Soares KC Rucki AA Kim V Foley K Solt S Wolfgang CL. TGF-Beta Blockade Depletes T Regulatory Cells From Metastatic Pancreatic Tumors in a Vaccine Dependent Manner. Oncotarget (2015) 6(40):43005–15. doi 10.18632/oncotarget.5656 PMC4767487 26515728

[57] Biffi G Oni TE Spielman B Hao Y Elyada E Park Y. IL1-Induced JAK/STAT Signaling Is Antagonized by TGFbeta to Shape CAF Heterogeneity in Pancreatic Ductal Adenocarcinoma. Cancer Discov (2019) 9(2):282–301. doi 10.1158/2159-8290.CD-18-0710 30366930 PMC6368881

[58] Zhang Y Lazarus J Steele NG Yan W Lee HJ Nwosu ZC. Regulatory T-Cell Depletion Alters the Tumor Microenvironment and Accelerates Pancreatic Carcinogenesis. Cancer Discov (2020) 10(3):422–39. doi 10.1158/2159-8290.CD-19-0958 PMC7224338 31911451

[59] Trebska-McGowan K Chaib M Alvarez MA Kansal R Pingili AK Shibata D. TGF-Beta Alters the Proportion of Infiltrating Immune Cells in a Pancreatic Ductal Adenocarcinoma. J Gastrointest Surg (2022) 26(1):113–21. doi 10.1007/s11605-021-05087-x PMC12289350 34260016

[60] Squair JW Gautier M Kathe C Anderson MA James ND Hutson TH. Confronting False Discoveries in Single-Cell Differential Expression. Nat Commun (2021) 12(1):5692. doi 10.1038/s41467-021-25960-2 34584091 PMC8479118

[61] Qiu P. Embracing the Dropouts in Single-Cell RNA-Seq Analysis. Nat Commun (2020) 11(1):1169. doi 10.1038/s41467-020-14976-9 32127540 PMC7054558

[62] Vayrynen SA Zhang J Yuan C Vayrynen JP Dias Costa A Williams H. Composition, Spatial Characteristics, and Prognostic Significance of Myeloid Cell Infiltration in Pancreatic Cancer. Clin Cancer Res (2021) 27(4):1069–81. doi 10.1158/1078-0432.CCR-20-3141 PMC8345232 33262135

[63] Rao A Barkley D Franca GS Yanai I. Exploring Tissue Architecture Using Spatial Transcriptomics. Nature (2021) 596(7871):211–20. doi 10.1038/s41586-021-03634-9 PMC8475179 34381231

[64] Vance K Alitinok A Winfree S Jensen-Smith H Swanson BJ Grandgenet PM. Machine Learning Analyses of Highly-Multiplexed Immunofluorescence Identifies Distinct Tumor and Stromal Cell Populations in Primary Pancreatic Tumors. Cancer Biomark (2022) 33(2):219–35. doi 10.3233/CBM-210308 PMC9278645 35213363

[65] Khened M Kori A Rajkumar H Krishnamurthi G Srinivasan B. A Generalized Deep Learning Framework for Whole-Slide Image Segmentation and Analysis. Sci Rep (2021) 11(1):11579. doi 10.1038/s41598-021-90444-8 34078928 PMC8172839

[66] Moncada R Barkley D Wagner F Chiodin M Devlin JC Baron M. Integrating Microarray-Based Spatial Transcriptomics and Single-Cell RNA-Seq Reveals Tissue Architecture in Pancreatic Ductal Adenocarcinomas. Nat Biotechnol (2020) 38(3):333–42. doi 10.1038/s41587-019-0392-8 31932730

[67] Danaher P Kim Y Nelson B Griswold M Yang Z Piazza E. Advances in Mixed Cell Deconvolution Enable Quantification of Cell Types in Spatial Transcriptomic Data. Nat Commun (2022) 13(1):385. doi 10.1038/s41467-022-28020-5 35046414 PMC8770643

[68] Stuart T Butler A Hoffman P Hafemeister C Papalexi Mauck WM 3rd.. Comprehensive Integration of Single-Cell Data. Cell (2019) 177(7):1888–902.e21. doi 10.1016/j.cell.2019.05.031 31178118 PMC6687398

